# Maritime cybersecurity: are onboard systems ready?

**DOI:** 10.1080/03088839.2022.2124464

**Published:** 2022-09-16

**Authors:** Kamlesh Kanwal, Wenming Shi, Christos Kontovas, Zaili Yang, Chia-Hsun Chang

**Affiliations:** a Liverpool Logistics, Offshore and Marine Research Institute (LOOM), Liverpool John Moores University, Liverpool, UK; b Maritime and Logistics Management, Australian Maritime College, University of Tasmania, Newnham, TAS 7248, Australia

**Keywords:** Cybersecurity, maritime security, human factor, cybersecurity compliance monitoring, structural equation modelling

## Abstract

Recent maritime cybersecurity accidents reveal that shipping is facing increased exposure to cyber threats, especially due to the fast-growing digitalisation of the sector, leaving vessels and their onboard systems vulnerable to cyberattacks. This research aims at evaluating the relationship among the critical dimensions influencing cybersecurity performance in the maritime industry. To achieve this, six critical dimensions related to cybersecurity preparedness are first identified through literature review, namely ‘regulations’, ‘company procedures’ from a managerial perspective and ‘shipboard systems readiness’, ‘training and awareness’, ‘human factor’ and ‘compliance monitoring’ at an operation level. A Likert-scale questionnaire is designed and used to collect empirical data from 133 seafarers and shore-based staff. Structural Equation Modelling (SEM) is applied to examine the causal relationships between the six dimensions and shipboard cybersecurity performance. The results show that ‘regulations’ positively influence shipping companies’ cybersecurity-related ‘procedures’, which in turn positively affects ‘shipboard systems readiness’, ‘training and awareness’, and ‘monitoring’. Further, ‘training and awareness’ positively influences the cybersecurity performance of ships. The results have profound implications for the shipping industry on how to strengthen their cyber practices in order to improve their cybersecurity performance. Recommendations for future academic research related to maritime cybersecurity are also provided.

## 1. Introduction

Cybercrime is recognised as one of the most significant threats any company would face in the next decades. According to Cybersecurity Ventures (2020), the costs of global cybercrime are expected increasing 15% per year between 2021 and 2025, and reaching USD 10.5 trillion by 2025, compared to USD 3 trillion in 2015. The introduction of automation, integration and the drive for digitalisation of systems and processes for optimising management processes makes ships increasingly prone to cyberattacks (Kala and Balakrishnan 2019).

A cyber incident poses a major risk to the maritime sector, which might lead to severe consequences in terms of human casualties, economic and environmental ramifications (Alcaide and Llave 2020). Cyberattacks are, therefore, considered important threats to the maritime industry. Vulnerabilities in hardware, software and network systems are exploited by cybercriminals, for example, by introducing malware into the victim’s equipment. The motive behind such attacks can be manifold and not limited to data theft, financial gains, disruption of service, obtaining intelligence, gaining media attention and political vendetta (CaproCaprolu et al. 2020; Kala and Balakrishnan 2019). Large companies (e.g. Maersk Line, BW group, COSCO) have witnessed serious attacks in the past, resulting in data breaches and significant commercial losses (Sakar et al. 2019). Even the global maritime regulatory body, the United Nations’ International Maritime Organization (IMO) was cyberattacked in September 2020 (Kuhn, Bicakci, and Shaikh 2021).

Some research has been conducted on cybersecurity issues in the maritime industry focusing mainly on hazards/threats influencing cybersecurity (e.g., Chang et al. 2019), policies and measures (Tam and Jones 2018), and training of maritime professionals (Tam, Moara-Nkwe and Jones 2020). However, there is a lack of sufficient analysis on the relationship between the critical factors and cybersecurity performance in the maritime industry, and it is widely recognised that such a relationship analysis is essential for effective risk control/management (e.g., Yang et al. 2018). To that extent, this work aims at evaluating the relationship between the critical dimensions and cybersecurity performance in the maritime sector. The key dimensions refer to the recognised groups of crucial risk factors influencing ship onboard cybersecurity performance (Chang et al. 2019).

This research adopts the ‘Diffusion of innovations’ theory to explain how the novel concept of cybersecurity is adopted by the relatively conservative maritime industry. Six critical dimensions that influence maritime cybersecurity performance are discussed in the literature review section, including ‘regulatory framework’, ‘company procedures’ from a managerial perspective, and ‘shipboard systems readiness’, ‘cyber training and awareness’, ‘compliance monitoring’ and ‘human factor’ at an internal operational level. A Likert-scale questionnaire is designed for collecting empirical data from 133 seafarers and onshore shipping company staff. This research further applies Structural Equation Modelling (SEM) to analyse the interrelationship amount the factors and cybersecurity performance in the maritime sector. The contributions of this research include: (1) provision of useful insights on the important factors that affect cybersecurity performance on ships, which could be further explored in order to identify measures to enhance maritime cybersecurity; (2) a new definition of maritime cybersecurity performance, as well as a novel causal relationship framework for modelling maritime cybersecurity factors and performance; (3) a number of cybersecurity policy recommendations for the interested parties.

The rest of this paper is structured as follows. Section 2 provides a literature review by identifying the critical dimensions affecting the cybersecurity performance of ships. Section 3 describes the research methodology and the hypotheses. The study findings and discussion are presented in Sections 4 and 5 respectively, the conclusions are drawn in Section 6.

## 2. Literature review

### 2.1. Diffusion of innovations theory

‘Diffusion of innovations’ theory, proposed by Rogers (1995), addresses how the public accepts new innovations (e.g., technologies, concepts, products). The process of acceptance of innovation includes the following five steps: awareness, interest, evaluation, trial and adoption. The theory has been applied in various areas such as mobile applications (Min, So, and Jeong 2019), environmental construction (Sartipi 2020), transportation (Nordhoff et al. 2021; Wang, Douglas, and Hazen 2021), and cybersecurity (Miron and Muita 2014). Our study is in line with this theory because the maritime industry has a long history of using established techniques as the best practice. Compared to other main classical transport modes (e.g., aviation), it often sits back in terms of accepting innovative technologies, especially for addressing safety and security that are often driven by accidents in a reactive way.

### 2.2. Critical factors influencing maritime cybersecurity

The section will elaborate on the critical factors influencing the cybersecurity of ships. Based on a thorough survey of the relevant literature, the following six critical dimensions are identified, reviewed, and categorised as the fundamentals affecting the cybersecurity performance of ships. Their definitions, justification of selection and supporting references are described in detail in the ensuing sections.

#### 2.2.1. Regulatory framework

The International Maritime Organisation (see IMO document MSC-FAL.1/Cir. 3) provides recommendations for addressing the cyber risks associated with the industry and developing and implementing best practices through the company’s safety management system (CaproCaprolu et al. 2020). The guidelines follow the five-step framework of the United States National Institute of Science and Technology (NIST) i.e. identify, protect, detect, respond and recover and have become mandatory for all ships following the company’s first annual verification of the Document of Compliance (DOC) after January 1^st^, 2021 (Kala and Balakrishnan 2019). Its compliance will be inspected by Port State Control officers (Wingrove 2021).

Many other entities such as classification societies, associations and unions have developed individual guidelines for the protection of ships, ports and connected organisations from cyber-crimes. For instance, BIMCO proposes a guideline for shipboard Information Technology (IT) and Operational Technology (OT) systems for the identification of threats and vulnerabilities, their assessment, development of mitigation and contingency measures, and responding and recovering from such threats (BIMCO, CLIA, ICS, Intercargo, Intermanager, Intertanko, IUMI, OCIMF and World Shipping Council 2020). American Bureau of Shipping (American Bureau of Shipping (2016)) presents guidelines for marine and offshore operations on cybersecurity, best practices, criteria for assessment of systems/assets and certification, concepts of data integrity, software systems verification and quality management. Lloyd’s Register (2016) provides guidelines for stakeholders on the design, installation, integration and operation of digitally enabled systems onboard ships and marine platforms to understand the implications of technology in digital systems. DNV-GL (2016) also provides guidance on the application of standards such as ISO/IEC 27001 and ISA-99/IEC-62443 (standard for OT security of industrial control systems such as Global Positioning System (GPS)).

#### 2.2.2. Cyber security-related company procedures

In compliance with the IMO (2017) guidelines, plans and procedures will need to be devised, taking into consideration the relevant standards, and guidelines to ensure the safety of ships/crew and the protection of the marine environment.

An effective company cybersecurity strategy would involve designing a policy in cooperation with the organisation and the stakeholders, and addressing key areas such as risk management, resource management, strategic alignment with the organisation, performance measurement, value delivery and integration with security. This will encompass the identification of key systems with respect to safety, operations and environmental protection and the level of acceptable risk for these (Kala and Balakrishnan 2019). The requirements as per the IMO’s International Safety Management (ISM) Code and the International Ship and Port Facility Security (ISPS) Code should now be matched with companies’ plans and procedures for cyber risk management.

#### 2.2.3. Ship’s systems readiness

IT and OT systems onboard ships have been integrated through network connectivity and the internet for improving performance, making them prone to cyberattacks. Attackers could hack the cyber-enabled systems through loosely secured network connections and bypass firewalls to disrupt services in order to steal data for selling or ask for ransom, to facilitate the illegal movement of cargo, to gather intelligence and knowledge of critical systems/infrastructure, for political gains, and many more, including even the use of the vessel as a weapon to attack other potential targets (CaproCaprolu et al. 2020).

It is also possible to undertake an attack on essential navigation equipment such as Automatic Identification System (AIS), Electronic Chart Display and Information System (ECDIS), GPS, Voyage data recorder (VDR) through jamming and. The disruption of these systems can result in severe consequences (e.g., collision or grounding) and may lead to human losses and/or pose a danger to the environment. IHS Fairplay (2016) conducts a survey and finds that several onboard systems including shipboard systems including GPS, ECDIS and Engine Control are very vulnerable to attacks.

Outdated software systems also pose a tangible cybersecurity risk and lack of timely application of patches/updates can also render the current systems vulnerable (Jones, Tam, and Papadaki 2016). Tam and Jones (2019) conduct a survey and disclose that 79% of the participants feel that shipboard computers and their internet activity are the weakest links. Sakar et al. (2019) study 14 Tukey-based shipping companies using semi-structured interviews to gain some understanding of their preparedness both ashore and onboard ships for dealing with cyber risks. They reveal that the shipboard IT systems are not fully protected against cyber threats and required investments for improvement.

#### 2.2.4. Cyber training and awareness

Recent cyber incidents in the maritime industry have revealed that many employees are not trained to respond appropriately to cyber threats, potentially leading to behaviour that does not contribute much to minimising risks and managing the issue (DNV-GL 2016). Previous studies have stated that cyber training raises awareness which in turn enhances cybersecurity (Bolat and Kayisoglu 2019; El-Bably 2021). Through comprehensive and regular training, sea crews would have awareness of what activities may cause cyberattacks and sufficient knowledge to mitigate the impact of being cyberattacked.

IMO doc. MSC-FAL.1/Circ.3 stated that there is an urgent need to raise awareness to have effective maritime cyber management. Tam and Jones (2019) analysed the factors affecting security and individual training levels, and find that most of the respondents consider the standards of crew training as the biggest issue. Svilicic et al. (2019) also found that the likelihood of lack of training and awareness to seafarers is the highest among their identified cyber threats. To avoid cyber assaults and to prepare both the crew and on-shore personnel to deal with such threats, training and awareness in cybersecurity-related matters is required (Chang et al. 2019). Training can be imparted to the company IT professionals for raising the defence skills, and the ship’s crew who lack the expertise and the background but play a crucial role in emergency and crisis management (Tam, Moara-Nkwe, and Jones 2020). Overall, crew awareness and readiness can constitute the primary barrier against any kind of cyberattack guided on ships (Alcaide and Llave 2020).

#### 2.2.5. Compliance monitoring

To comply with the IMO’s cyber regulations, organisations need to determine their cybersecurity priorities by conducting a risk assessment to identify risks and weaknesses specific to their ships and, then, adopt effective cyber control measures and industry best practices to mitigate them (Whitterker 2016). Enforcement of the IMO’s Guidelines will be verified by Port State Control officers in a way that the ships do not only need to carry the required certificates and documents but also to prove through their procedures a good understanding of cyber risks. Failure to appropriately address cyber risks as per the Guidelines would result in non-compliance, fines and other actions that could follow based on the identified deficiencies (Wingrove 2021).

Regular internal and external audits can assist in re-evaluating the cybersecurity measures in place and provide an early indication of loopholes and weaknesses in the system. Any pitfalls could be bridged timely to maintain efficient and sound security practices.

#### 2.2.6. Human factor

Around 80% to 90% of maritime accidents are attributed to human factors (Macrae 2009; Heij and Knapp 2018; Chang et al. 2021). Humans are seen as the weakest link in the management of cybersecurity and lack of knowledge and awareness on their part can result in human errors (Boyce et al. 2011; El-Bably 2021). According to an IBM report, 95% of cyber security breaches are caused by human error and may result in unauthorised access through a secure door or barrier, the so-called ‘tailgating’ (IBM 2014). Such breaches may cause data loss, disruption of service or even using ships as a potential weapon and can therefore result in economic losses and/or environmental damages.

The modern-day sophisticated systems onboard vessels are operated by a staff who are rotated on a variety of ships with different systems thereby making them unfamiliar and prone to errors in the management of security breaches (Hopcraft and Martin 2018). A number of cybersecurity-related human errors can be linked to any of the following activities such as accessing suspicious websites or links or disabling firewalls due to carelessness or for a specific purpose, using personal devices on shipboard systems, etc.

### 2.3. Cybersecurity performance

The term ‘cybersecurity performance’ is in general described as the security performance of an entity from a cyber perspective. It has been used in the academic literature. For instance, Lee (2021) proposed a new framework for cyber risk management that includes four layers: cyber ecosystem, cyber infrastructure, cyber risk assessment and cyber performance, which includes three activities (i.e., implementation, monitoring and controlling, and continuous improvement). Garcia-Perez, Sallos, and Tiwasing (2021) defined four indicators for cybersecurity performance, including Focus (provision of foundational cybersecurity element), Capability, Resilience and Prep (cybersecurity training and preparation efforts). However, there is no universally accepted definition of cybersecurity performance. Through the description of cybersecurity performance from the existing research, we propose the following definition of maritime cybersecurity performance: *the effectiveness and efficiency of a range of actions taken to prevent or mitigate the impact of cyberattacks on OT/IT systems of ships, as well as the financial performance and reputation of companies*.

We have identified five main ship systems, which need protection against cybercrimes, namely, navigation systems, plant and machinery, communication systems, OT, and IT systems. Loss or compromise of any of these systems can impact the operational capability and efficiency and jeopardise the safety of their ship and staff (Boyes and Isbell 2017). Cybercrimes affect the safety of navigation by exploiting the vulnerabilities of some of the essential navigation equipment such as AIS, GPS, ECDIS, VDR, and communication systems. These systems can be hacked, jammed, spoofed and servicse can be disrupted, which can lead to disastrous consequences such as collisions and groundings etc. Such incidents would not only lead to severe economic losses but also result in environmental pollution, which can in turn damage habitats and ecosystems. Other cases may involve the breach of systems to manipulate data such as cargo manifests in order to carry out a theft of cargo or obtain the client’s confidential information. Such devastating effects of cybercrimes can damage the reputation of shipping companies and result in further business disruption.

Considering the growing threat of cybercrimes on ships and their devasting consequences on the reputation and the financial performance of shipping companies, an indepth study is needed to enhance the cybersecurity performance of ships by focusing on efficient cybersecurity practices. To address it, new research hypotheses are proposed in Section 2.4.

### 2.4. Research hypotheses

To examine how the above six dimensions affect the overall cybersecurity performance of ships, nine hypotheses are proposed in this paper.

Adoption of a cyber regulatory framework and guidelines can ensure that organisations develop a robust cybersecurity management system to address cyber threats using proven risk management principles to prioritize security activities and performance within the organisation (Belmont 2014). Hence, the following hypothesis is proposed:

***H1—Regulatory framework positively impacts company procedures***

Effective company cyber procedures may improve cybersecurity through several internal operational factors. Shipping companies can utilise specific security procedures and policies for the management of digital/technology enabled systems and networks to enhance the resilience and robustness of the systems (BIMCO, CLIA, ICS, Intercargo, Intermanager, Intertanko, IUMI, OCIMF and World Shipping Council 2020). Moreover, effective policies and procedures can also enhance the cybersecurity awareness of staff by addressing training requirements based on their roles (Bolat and Kayisoglu 2019). Procedures can impact company training plans and further enhance staff cybersecurity awareness. In addition, a proper cybersecurity procedure can provide a clear guideline for the requirement of cybersecurity monitoring. Monitoring, reviewing and auditing requirements of cyber critical ship systems and processes are defined by the organisational procedures and plans (Boyes and Isbell 2017; DNV-GL 2016). This includes verification of controls and identification of gaps for ensuring enhanced protection against such crimes. Besides, straightforward cybersecurity procedures can reduce human errors and prevent their negative impacts (Chang et al. 2019). Hence, the following hypotheses are proposed:

***H2—Cyber procedures have a positive effect on the resilience and robustness of ship’s systems***

***H3—Cyber procedures have a positive impact on cyber training and awareness***

***H4—Cyber procedures have a positive impact on compliance monitoring***

***H5—Cyber procedures have a negative impact on the occurrence of human factor***

On the other side, proper implementation of internal operations can lead to better cybersecurity performance. The resilience of shipboard systems is closely linked to the vessel’s safety and operational capability (Boyes and Isbell 2017). Systems with up-to-date firewalls and antivirus software to deal with cyberattacks can largely improve cybersecurity performance and further enhance the company’s competitiveness (Chang et al. 2019). Moreover, with the growing use of technology in shipboard functions, a lack of cyber-related training and staff/seafarers’ cybersecurity awareness becomes detrimental. Training can help respond to cyber risks more proactively and enhance safety and security (Chang et al. 2019; Tam, Moara-Nkwe, and Jones 2020). In addition, regular monitoring and inspections of network components can help organisations to analyse their cybersecurity measures and further mitigate the impact of cybersecurity risk (Pandey et al. 2020). In addition, information security breaches related to human factors can result in serious consequences such as losses of revenue, productivity, reputation and competitive advantage (Chang et al. 2019). This leads us to the following hypotheses:

***H6—Resilience of onboard systems has a positive impact on ship’s cybersecurity performance.***

***H7—Cyber training and awareness has a positive impact on ship’s cybersecurity performance.***

***H8—Cyber compliance monitoring has a positive impact on ship’s cybersecurity performance***

***H9—Human factor has a negative impact on ship’s cybersecurity performance***

**Figure 1.**
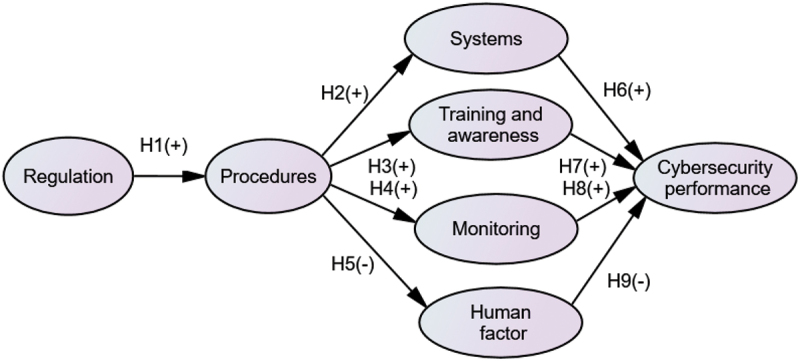
Conceptual research framework.

A conceptual model to illustrate the relationship of the above nine hypotheses is shown in Figure 1.

## 3. Methodology

### 3.1. Data collection

To ensure collecting adequate representation for the research, a non-probability convenience sampling combined with a snowball sampling technique is used. The target sample for the study is seafarers and shipping company staff, who are directly responsible for implementing and monitoring cybersecurity measures on ships. A five-point Likert scale (1: Strongly Disagree, 2: Disagree, 3: Neutral, 4: Agree, 5: Strongly Agree) questionnaire has been utilised through an online survey tool and a total of 150 questionnaires are sent out to ensure an unbiased response and an adequate representation in May 2021. The academic literature that inspired the question item design is listed in Appendix A. The main reasons for using an online survey are its advantages of easiness of administration and analysis, and to capture more respondents (including seafarers who have busy and irregular schedules, and are spread all across the world).

### 3.2. Analysis method

First, we conduct a descriptive analysis using SPSS 27 to derive the respondents’ perception towards the cybersecurity readiness of ships. Reliability and validity tests are performed using the Composite Reliability (CR) and Average Variance Extracted (AVE) metrics. CR and AVE values are calculated using Equations (1–2) respectively (Chang et al., 2021):

(1) $$C.R.=\frac{{\left(\sum_{1}^{n}\lambda \right)}^{2}}{{\left(\sum_{1}^{n}\lambda \right)}^{2}+\sum_{1}^{n}\left(1-\lambda^{2}\right)}$$

(2) $$\mathrm{AVE}=\frac{\sum_{1}^{n}\lambda^{2}}{\sum_{1}^{n}\lambda^{2}+\sum_{1}^{n}\left(1-\lambda^{2}\right)}$$

where λ is factor loading and n is the number of items under each dimension.

Confirmatory Factor analysis (CFA) has also been performed for finding a direct and indirect correlation between the factors. Thereafter a Structural Equation Modelling (SEM) analysis is employed to test the specified hypotheses. SEM is a multivariate analysis for simultaneously investigating complex causal relationships among observable variables and latent variables, as well as between latent variables; see Kline (2016) for more on SEM. The observable variables are the question items, whereas the latent variables are the seven identified dimensions. Each dimension has several relevant questions. Compared to normal regression that can also analyse causal relationships, the advantage of SEM is the ability to simultaneously investigate the whole model with both measurement and structural considerations (Kao, Stewart, and Lee 2009). SEM has been widely applied in the maritime research domain; see for example Lu, Shang, and Lin (2016), Lu et al. (2020)), Lin and Chang (2021).

Through this SEM analysis, the nine proposed hypotheses will be tested, and we can understand whether the managerial factors would influence internal operational ones, which further affect maritime cybersecurity performance. A number of fit indices are used to test the fitness of the model; see Table 4. We utilise the SEM and perform the analysis using the AMOS 27 statistical package.

## 4. Research findings

### 4.1. Respondent profile

A total of valid 133 replies are collected. Most of the respondents are seafarers (84.2%), while the remaining 15.8% are onshore personnel working for various shipping companies. Regarding their work experience, around half of the participants have a considerable experience (more than 16 years) in the maritime industry (50.4%), followed by 11 to 15 years (22.6%), 6 to 10 years (21%) and less than 5 years (6%). These indicate that the respondents can provide valuable opinions on this topic as they have adequate and relevant experience.

### 4.2. Results of descriptive analysis

The results of the descriptive analysis are shown in Table 1. Overall, the identified six cybersecurity-related dimensions have a neutral to high agreement response with the overall cybersecurity readiness (mean between 3 and 4). The highest factor is ‘Procedures’ (mean: 3.94), followed by ‘Systems’ (mean: 3.90), ‘Monitoring’ (mean: 3.85), ‘Training and awareness’ (mean: 3.79), ‘Regulations’ (mean: 3.58), and ‘Human factor’ (mean: 3.36). Apart from the six dimensions, the overall cybersecurity performance of ships is currently high (mean: 4.26), which indicates that most of the respondents agree that shipping companies could have better performance through the above cybersecurity implementations. Among the identified items, four have mean values higher than 4, see items Pro3, Tra4, Sys2, and Sys3 in Table 1.

**Table 1.** Respondents’ perceptions of the critical dimensions and performance.

Question Items	Code	Mean	S.D.
***Regulation***		3.58	0.66
The current regulations for maritime cybersecurity are easy to understand and have clarity.	Reg1	3.72	0.70
The requirements for maritime cybersecurity are specific and detailed i.e. cover the necessary areas.	Reg2	3.62	0.81
The requirements are easy to apply to existing navigation/transaction operations.	Reg3	3.41	0.89
***Procedures***		3.94	0.78
Our company procedures are specific and detailed and cover all aspects of cybersecurity in accordance with the required regulations.	Pro1	3.89	0.83
Third-party access to systems has been properly and appropriately addressed by our company procedures.	Pro2	3.86	0.94
Our company policy on the use of personal devices on the systems is clear and appropriate.	Pro3	4.08	0.91
The procedures are reviewed and updated by our company at regular intervals for addressing new vulnerabilities.	Pro4	3.93	1.02
***Systems***		3.90	0.80
The IT and OT systems/equipment are designed and maintained to provide maximum protection i.e. are difficult to breach	Sys1	3.59	0.97
The latest firewall and antivirus are used for the systems.	Sys2	4.02	0.89
The software for the systems is kept up to date at all times.	Sys3	4.02	1.03
Patches to the systems are applied timely.	Sys4	3.97	0.93
***Training and awareness***		3.79	0.88
Our company regularly holds maritime cybersecurity training.	Tra1	3.73	1.07
Cybersecurity training that is carried out by our company covers all critical aspects of shipboard/shore security.	Tra2	3.70	1.07
I have received appropriate training in cybersecurity.	Tra3	3.65	1.07
I am aware of the various cyber risks on ships.	Tra4	4.03	0.86
I am aware of the procedures to follow in case of a cyberattack.	Tra5	3.85	0.98
***Human factor***		3.36	0.84
It is possible that the staff bypass firewalls for specific purposes or due to a lack of awareness.	Her1	3.19	1.16
It is possible that the staff access suspicious websites and links due to careless operations or for a specific purpose.	Her2	3.40	1.04
The awareness of not using personal devices on systems is low.	Her3	3.33	0.94
Third-party removable media is not always scanned before inserting in shipboard/shore systems.	Her4	3.47	1.07
It is possible that shipboard/shore sensitive information is shared via social media.	Her5	3.42	1.01
***Monitoring***		3.85	0.76
Cyber compliance with the statutory and company procedures is regularly monitored by a responsible officer on our ships/ashore.	Mon1	3.84	0.87
Internal cyber audits are regularly conducted by our company representative to verify any loopholes.	Mon2	3.82	0.93
Our company ships/offices are frequently inspected by parties such as Port State Control officers/oil major companies/third parties for cybersecurity preparedness.	Mon3	3.90	0.94
***Cybersecurity Performance***		4.26	0.69
Cybersecurity implementation can largely improve the safety/security of ships/ shipping companies and prevent cybercrimes.	Per1	4.29	0.74
Cybersecurity implementation can help ships/shipping companies have a better reputation.	Per2	4.21	0.79
Cybersecurity implementation can help ships/shipping companies have a better financial performance.	Per3	4.29	0.85

### 4.3. Results of CFA

**Figure 2.**
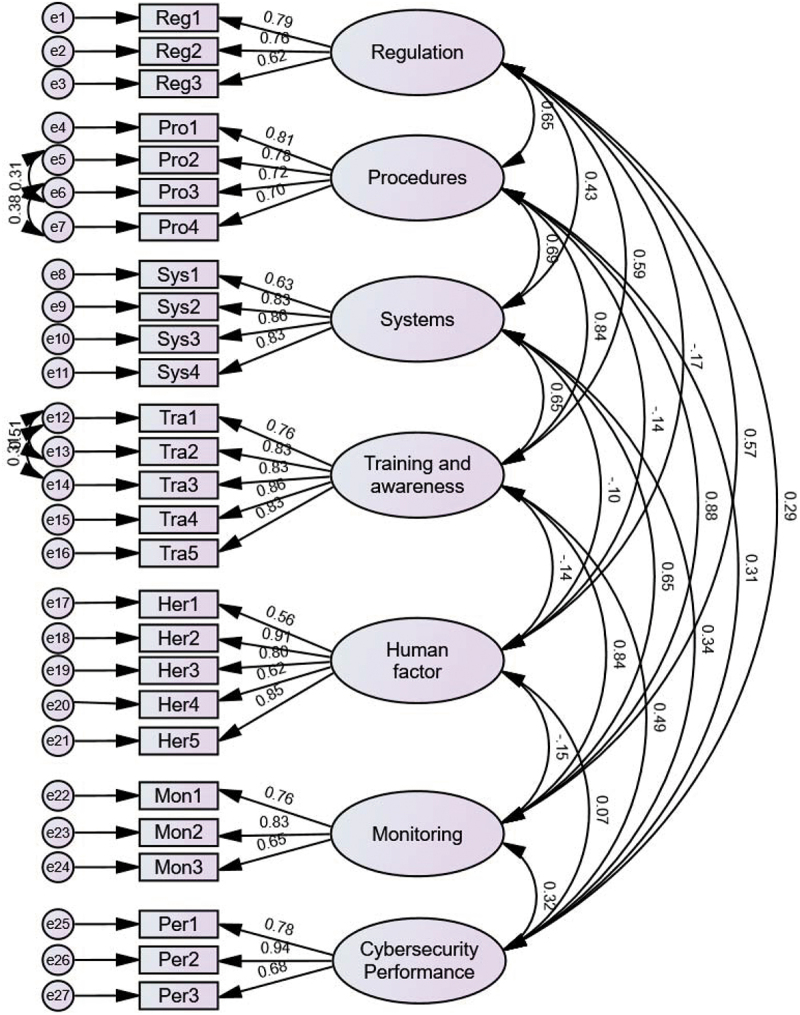
CFA with standardised estimates.

CFA is conducted to verify the proposed structure, the results of which are presented in Figure 2. Several statistical tests are used to determine how well the model fits the data; see Table 2 for the metrics used and the fitness criteria. All metrics meet the recommended criteria.

**Table 2.** Results of CFA.

Metric	Value	Criteria
Chi-square/degrees of freedom (CMIN/df)	1.51	< 3.0
Comparative Fit Index (CFI)	0.93	> 0.9
Goodness of Fit (GFI)	0.81	> 0.8
Tucker-Lewis Index (TLI)	0.92	> 0.9
Incremental Fit Index (IFI)	0.93	> 0.9
Root Mean Square Residual (RMR)	0.06	< 0.1
Root means square of approximation (RMSEA)	0.06	< 0.08

### 4.4. Reliability and validity tests

Table 3 shows the results of the reliability and validity tests for the proposed model. The factor loadings $\left(\lambda \right)$ for the variables are in the range between 0.56 and 0.9, more than the recommended value of 0.5, indicating a good relationship between the latent construct and the observed variables. It also confirms that the variance of the variables is sufficient for extraction by each dimension. The CR and AVE values are well above the recommended thresholds of 0.7 and 0.5 respectively (see Fornell and Larcker (1981) for more), implying that the model meets the reliability and validity tests.

**Table 3.** Reliability and validity test results.

Research Item	Factor Loadings $\left(\lambda \right)$	Error	Composite Reliability (CR)	Average Variance extracted (AVE)
***Regulation***			**0.83**	**0.53**
Reg1	0.79	0.19		
Reg2	0.76	0.27		
Reg3	0.62	0.48		
***Procedures***			**0.89**	**0.63**
Pro1	0.75	0.30		
Pro2	0.83	0.28		
Pro3	0.82	0.26		
Pro4	0.77	0.42		
***Systems***			**0.88**	**0.63**
Sys1	0.63	0.56		
Sys2	0.83	0.24		
Sys3	0.86	0.27		
Sys4	0.83	0.26		
***Training and Awareness***			**0.91**	**0.68**
Tra1	0.76	0.47		
Tra2	0.83	0.35		
Tra3	0.83	0.35		
Tra4	0.86	0.20		
Tra5	0.83	0.29		
***Human factor***			**0.85**	**0.58**
Her1	0.56	0.91		
Her2	0.91	0.18		
Her3	0.80	0.32		
Her4	0.62	0.70		
Her5	0.85	0.28		
***Monitoring***			**0.82**	**0.57**
Mon1	0.76	0.32		
Mon2	0.83	0.26		
Mon3	0.66	0.50		
***Cybersecurity Performance***			**0.89**	**0.65**
Per1	0.78	0.21		
Per2	0.94	0.08		
Per3	0.68	0.39

**Table 4.** Results of fitness tests.

Metric	Value	Threshold
Chi-square/degrees of freedom (CMIN/df)	1.47	< 3.0
Comparative Fit Index (CFI)	0.93	> 0.9
Goodness of Fit (GFI)	0.81	0.8–0.9
Normed Fit Index (NFI)	0.82	0.8–0.9
Tucker-Lewis Index (TLI)	0.93	> 0.9
Incremental Fit Index (IFI)	0.94	> 0.9
Root Mean Square Residual (RMR)	0.05	< 0.1
Root means square of approximation (RMSEA)	0.06	< 0.08

### 4.5. Results of SEM

**Figure 3.**
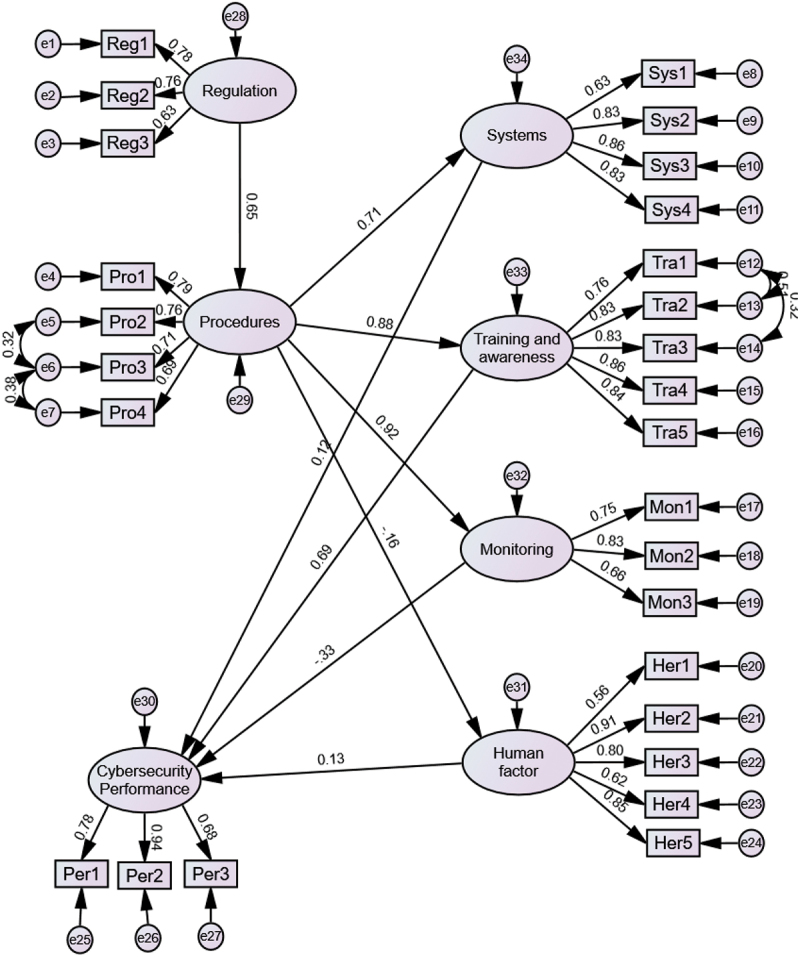
SEM results with standardised estimates.

The SEM model with the standardised estimate results is shown in Figure 3. The results of the model fitness parameters are presented in Table 4. Overall, the model meets the recommended criteria indicating a good fit.

Table 5 shows the results of SEM. Based on a threshold p-value of 0.1, the results show that ‘Regulations’ has a positive and significant impact on company ‘Procedures’ (supporting H1); ‘Procedures’ also has a significant and positive impact on ‘Systems Readiness’, ‘Training and awareness’ and ‘Monitoring’ (supporting H2, H3 and H4, respectively). However, ‘Procedures’ does not have a significant and negative impact on the occurrence of ‘Human factor’ (H5 is not supported). Besides, ‘Training and awareness’ has a significant positive impact on ‘Performance’ (supporting H7). However, the results do not show a significant impact of ‘Monitoring’, ‘Systems’ and ‘Human factor’ on ‘Performance’ (not supporting H6, H8 and H9).

**Table 5.** Results of SEM.

			Estimate	S.E.	C.R.	P	Results
Procedure	<–	Regulation	0.741	0.147	5.055	***	Supports H1
Systems	<–	Procedure	0.994	0.144	6.883	***	Supports H2
Training and awareness	<–	Procedure	1.246	0.154	8.088	***	Supports H3
Monitoring	<–	Procedure	0.892	0.134	6.666	***	Supports H4
Human factor	<–	Procedure	−0.189	0.115	−1.639	0.101	Not Support H5
Performance	<–	Systems	0.081	0.083	0.982	0.326	Not Support H6
Performance	<–	Training and awareness	0.45	0.129	3.494	***	Supports H7
Performance	<–	Monitoring	−0.314	0.195	−1.614	0.106	Not Support H8
Performance	<–	Human factor	0.1	0.067	1.505	0.132	Not Support H9

## 5. Discussion

### 5.1. Current level of cyber preparedness in the maritime industry

Due to the growing widespread concern about cyber-crimes, in 2017, the IMO adopted Resolution MSC.428(98) ‘Maritime Cyber Risk Management in Safety Management Systems’ and issued ‘Guidelines on Maritime Cyber Risk Management’ (see IMO doc. MSC-FAL.1/Circ.3), to provide guidance on how to contact an assessment of cyber risk through the shipping company’s safety management system (CaproCaprolu et al. 2020). A company safety management system needs to appropriately address the cyber risks by taking into consideration the relevant standards and guidelines to ensure the safety of ships/crew and the protection of the marine environment (BIMCO, CLIA, ICS, Intercargo, Intermanager, Intertanko, IUMI, OCIMF and World Shipping Council 2020). Despite this general guidance, there is a lack of a clear description on how to develop such a safety management system in shipping companies for cybersecurity, in part because the causal relationship between key influential dimensions/factors and cybersecurity performance remains unclear. Shipping companies have therefore complied with the IMO cyber risk management requirements based on the best practice in the field, devised and implemented company-specific cyber procedures and policies for their ships. However, the issues on whether these procedures can adequately address the cybersecurity of onboard systems, maintenance of cyber-related equipment, training of their staff and compliance monitoring requirements still remain unclear. In addition, the procedures should be clear and easy to understand for the seafarers and staff, e.g., using flowcharts with concise descriptions to present the necessary activities to enhance maritime cybersecurity performance.

Shipboard systems with up-to-date anti-cyberattack designs are vital. For the mitigation of cyberattacks, onboard systems should be equipped with cybersecurity functions such as intelligent isolation and fast recovery, and also be regularly updated (Jones, Tam, and Papadaki 2016). For example, equipment such as ECDIS requires regular updates to ensure safe operation. These updates can be applied online or through a USB stick, both methods are prone to viruses.

Cybersecurity training is another key factor that affects cybersecurity performance. To enhance the cyber awareness and skills for mitigating cyber threats and thus enhancing the cybersecurity readiness, shipping companies need to conduct regular and up-to-date training in conjunction with sound cybersecurity solutions such as the use of antivirus software, spam filters, firewalls, enforcement of complex passwords, etc. Besides, behaviour-based training needs to be implemented for the staff to prevent intentional errors. To change the behaviour of both offshore staff and onboard crew, shipping companies need to do much more than annual training. A solution could be to foster a cybersecurity culture that includes an effective engagement with sound and robust cybersecurity best practices. A refresher training should also be carried out at regular intervals by shipping companies to cater for the dynamic environment, change in requirements, new personnel etc. (DNV-GL 2016).

Monitoring is also crucial to enhance ship’s cybersecurity performance. There is a need to put in place stringent monitoring requirements to ensure that industry recommendations, company policies and best practices are being adhered to at all times.

Meanwhile, some gaps in cybersecurity in the form of human factor still exist. Certain unsafe practices such as using personal devices and accessing suspicious websites are still prevalent in the industry; these can be exploited and infringed by criminals. This is in line with the findings reported in Alcaide and Llave (2020). Respondents accepted the possibility of indulging in unsafe activities such as accessing suspicious websites/links, using personal devices on shipboard systems and sharing sensitive information via social media. Third-party removable media are also being used on shipboard systems without scanning them first, thereby, putting the ship’s systems at risk.

### 5.2. Impact of the cyber critical factors on cybersecurity performance

Regulations have a significant positive impact on procedures. Hence, shipping companies must strictly adhere to the regulatory framework for developing efficient cybersecurity policies, procedures and plans. Many regulations have been proposed by various organisations (see Section 2.2.1). Cyber security-related procedures have a significant positive effect on the resilience of onboard systems, training and awareness, and monitoring. Shipping should set stringent cybersecurity procedures, which provide guidelines for seafarers on shipboard systems operations and for the requirements of shipboard systems in cyber protection such as applying web and email content filtering.

Training and awareness have a significant positive impact on cybersecurity performance. Therefore, organisations must identify and address specific cyber training requirements for their staff and ensure that this is performed timely and upgraded at regular intervals. The training should also be provided regularly (e.g., every year or every half year) to staff and seafarers. Shipping companies could provide industrial case studies and best practices and collaborate with maritime universities, which in turn could provide high-standard cyber training courses to students, new seafarers and shipping company staff, and thus raise higher cybersecurity awareness related, for example, to phishing emails and websites, and encourage using strong passwords and changing them regularly. Government or maritime related associations could also recommend, mandate or provide cybersecurity training courses with recognised certificates to motivate more seafarers and staff to take the training.

### 5.3. Implications

The implications of this research are discussed from three aspects: managerial, academic and policy recommendations.

For managerial implications, this research addresses how shipping companies and seafarers can benefit in a more cost-effective way since the most influencing factors have been identified to guide and prioritise the measures to improve cybersecurity performance. Given that shipping companies often have limited budgets to address safety and cybersecurity concerns, this is especially the case also for small enterprises, our findings are insightful for them as they can guide these companies on how to obtain a satisfactory cybersecurity performance in a cost-effective manner. By ranking the crucial factors, companies can understand which factors are important and more efficient to improve their cybersecurity performance.

Regarding academic implications, SEM has been applied in many fields but there are limited applications related to cybersecurity. It shifts a new paradigm on the analysis of critical factors and performance in different maritime domains and even in a wider range of applied contexts such as those suffering high level of cybersecurity risks (e.g., finance and military). Further, the SEM based study presented in this paper also paves the way for further studies on the rational development of cybersecurity risk control measures. Future research can be extended in two directions: a) modelling of the relationships between risk factors/dimensions and the joint impact of multiple factors on cybersecurity performance; and b) modelling of cybersecurity risk-based decision making by incorporating our findings with economic information of the identified cybersecurity measures.

This paper also provides some policy recommendations to enhance the cybersecurity performance of the industry and to help more seafarers and staff understand the importance of cybersecurity as well as the skills required for dealing with cyberattacks. Through the collaboration among industry, government and academics, maritime cybersecurity performance can be significantly and efficiently enhanced.

## 6. Conclusions

This paper evaluates the interrelationship between six cybersecurity dimensions and the cybersecurity performance of ships. The results suggest that the shipping sector has had a positive outlook on the cyber regulatory framework and shipping companies have developed and implemented specific cyber procedures through their safety management system. These policies adequately cover critical areas of shipboard cybersecurity such as systems readiness, training and monitoring requirements. The results also show that cyber regulations positively impact the organisational procedures, which further positively and significantly impact the systems, training and awareness, and monitoring requirements. In addition, training and awareness is also found to have a positive impact on the overall cybersecurity performance of ships. Hence, to address cyber threats and the weak links in current cybersecurity practices, the maritime industry needs to improve cyber risk awareness among both onboard and shore-based staff by conducting regular and up-to-date training.

Unlike previous research, which mainly focused on identifying cyber weaknesses in the maritime industry, this research focuses on assessing the cybersecurity preparedness of ships and explaining the link of critical dimensions of cybersecurity to the overall preparedness and performance of ships. To our knowledge, this is among the first papers addressing these aspects of maritime cybersecurity and, thus, provides new research directions for academic research.

This paper has some research limitations which can be the focus of future research. First, the research framework is based on a literature review. Future research could further streamline the questionnaire and the theoretical framework, while incorporating other aspects such as user behaviour based on the so-called ‘protection motivation theory’, the ‘technology acceptance model’ and the ‘diffusion of innovations theory’. Secondly, future work can focus on collecting a larger sample from multinational seafarers to get a more representative opinion of the industry and potentially to focus on cultural behaviour differences. In addition, an analysis of the respondents’ opinions based on their background (e.g., experience, position, company size) can enhance our understanding of the respondents’ professional experience, e.g., related to the size of the technology on board the ships and the role of training the personnel involved in the operational processes. Finally, apart from the factors discussed in this work, there are various variables that can also affect the overall cybersecurity performance such as education, position, leadership, culture, and some human factors-related aspects such as fatigue, mental health issues, frustration, cognitive workload (for more details see Paul and Dykstra 2017; Zăgan et al. 2018; Tam et al. 2021; Telschow and Neider 2021). Future research could include these variables into the structure in order to obtain a more comprehensive picture.

## Appendix A

**Table:**

Question Items	References
***Regulation***	
The current regulations for maritime cybersecurity are easy to understand and have clarity.	IMO (2017); Hopcraft and Martin (2018); Caprolu et al. (2020)
The requirements for maritime cybersecurity are specific and detailed i.e. cover the necessary areas.	
The requirements are easy to apply to existing navigation/transaction operations.	
***Procedures***	
Our company procedures are specific and detailed and cover all aspects of cybersecurity in accordance with the required regulations.	IMO (2017); Chang et al. (2019);
Third-party access to systems has been properly and appropriately addressed by our company procedures.	
Our company policy on the use of personal devices on the systems is clear and appropriate.	
The procedures are reviewed and updated by our company at regular intervals for addressing new vulnerabilities.	
***Systems***	
The IT and OT systems/equipment are designed and maintained to provide maximum protection i.e. are difficult to breach	Jones, Tam, and Papadaki (2016); Chang et al. (2019); Sakar et al. (2019); Tam and Jones (2019)
The latest firewall and antivirus are used for the systems.	
The software for the systems is kept up to date at all times.	
Patches to the systems are applied timely.	
***Training and awareness***	
Our company regularly holds maritime cybersecurity training.	Bolat and Kayisoglu (2019); Chang et al. (2019); Tam, Moara-Nkwe, and Jones (2020)
Cybersecurity training that is carried out by our company covers all critical aspects of shipboard/shore security.	
I have received appropriate training in cybersecurity.	
I am aware of the various cyber risks on ships.	
I am aware of the procedures to follow in case of a cyberattack.	
***Human factor***	
It is possible that the staff bypass firewalls for specific purposes or due to a lack of awareness.	Hopcraft and Martin (2018); Tam and Jones (2019); Chang et al. (2020)
It is possible that the staff access suspicious websites and links due to careless operations or for a specific purpose.	
The awareness of not using personal devices on systems is low.	
Third-party removable media is not always scanned before inserting in shipboard/shore systems.	
It is possible that shipboard/shore sensitive information is shared via social media.	
***Monitoring***	
Cyber compliance with the statutory and company procedures is regularly monitored by a responsible officer on our ships/ashore.	Whitterker (2016); Wingrove (2021)
Internal cyber audits are regularly conducted by our company representative to verify any loopholes.	
Our company ships/offices are frequently inspected by parties such as Port State Control officers/oil major companies/third parties for cybersecurity preparedness.	
***Cybersecurity Performance***	
Cybersecurity implementation can largely improve the safety/security of ships/ shipping companies and prevent cybercrimes.	Peng and Lu (2018); Chang et al. (2019); Tam, Moara-Nkwe, and Jones (2020)
Cybersecurity implementation can help ships/shipping companies have a better reputation.	
Cybersecurity implementation can help ships/shipping companies have a better financial performance.	
